# Molecular epidemiology of tuberculosis in Sicily, Italy: what has changed after a decade?

**DOI:** 10.1186/s12879-014-0602-4

**Published:** 2014-11-19

**Authors:** Celestino Bonura, Michel K Gomgnimbou, Guislaine Refrégier, Aurora Aleo, Teresa Fasciana, Anna Giammanco, Christophe Sola, Caterina Mammina

**Affiliations:** Department of Sciences for Health Promotion and Mother-Child Care “G. D’Alessandro”, University of Palermo, Palermo, Italy; CNRS-Université Paris-Sud, Institut de Génétique et Microbiologie, UMR8621, Infection Genetics Emerging Pathogen Evolution Team, Orsay, France

**Keywords:** Tuberculosis, Sicily, Epidemiology, Spoligotyping, MIRU-VNTR

## Abstract

**Background:**

We aimed to investigate the molecular epidemiology of *Mycobacterium tuberculosis* complex (MTBC) isolates in the province of Palermo, Sicily, Italy, by characterizing 183 isolates identified in the years 2004-2012. A comparison with 104 MTBC strains identified in the same geographic area in the years 1994-2000 was also carried out.

**Methods:**

One hundred eighty-three MTBC isolates identified in Palermo, Italy, in the years 2004-2012 were analyzed by spoligotyping and the 24 mycobacterial interspersed repetitive unit (MIRU)-variable-number tandem-repeat (VNTR) method typing. Susceptibility testing to streptomycin, isoniazid, rifampin and ethambutol was also performed. Furthermore, the spoligotyping dataset obtained from 104 MTBC isolates identified from 1994 to 2000 was reanalyzed. Distribution into lineages and clustering of isolates in the two periods was compared.

**Results:**

One hundred seventy-seven out of the 183 isolates of MTBC submitted to molecular typing were fully characterized. Of these, 108 were from Italian-born and 69 from foreign-born individuals. Eleven different lineages and 35 families-subfamilies were identified with the most represented lineages being Haarlem (26.5%), T (19.2%), LAM (13.6%) and S (8.5%). Except for the Haarlem lineage, where isolates from foreign-born patients were overrepresented, the distribution of isolates in the families belonging to the Euro-American clone reflected the proportions of the two subpopulations. A total of 27 (15.2%) strains were clustered and three clusters were mixed. Approximately 25% of the 183 MTBC isolates under study proved to be resistant to at least one antiTB drug, with only three isolates categorized as multidrug resistant (MDR). When MTBC isolates identified in the years 1994-2000 were reanalyzed, lineages T (30.8%), LAM (29.8%), Haarlem (16.3%) and S (13.5%) proved to be predominant. No MTBC isolates belonging to CAM, U, CAS, Turkish and Ural lineages were identified.

**Conclusions:**

A wide heterogeneity was detected among the MTBC strains isolated in the years 2004-2012. Six lineages were not present among the isolates of the period 1994-2000. Comparison between distribution of lineages in the two consecutive periods depicts rapid and deep changes in the TB epidemiology in Palermo, Italy. An universal and continued laboratory-based surveillance of TB in Sicily is required.

**Electronic supplementary material:**

The online version of this article (doi:10.1186/s12879-014-0602-4) contains supplementary material, which is available to authorized users.

## Background

Tuberculosis (TB) is still a prominent cause of morbidity and mortality all over the world. The epidemiology of TB in low endemic western countries is strongly influenced by immigration from high endemic countries [1],[2]. According with the more recent European Centre for Disease Prevention and Control (ECDC) surveillance report focusing the TB epidemiological situation, in 2012 the overall proportion of TB cases of foreign origin in the European Union/European Economic Area (EU/EEA) was 26.8% with some northern European countries showing rates as high as 85.4% [3]. An accurate assessment of the impact of this phenomenon is indispensable for planning adequate control and prevention strategies. However, this objective is increasingly proving to be more challenging than expected. Indeed, in low EU/EEA prevalence countries, TB is now predominant in hard-to-reach, vulnerable population groups, mainly including socially excluded individuals, such as prisoners, drug addicts, homeless persons, recent and illegal immigrants [3]. These subjects are at high risk to be diagnosed and treated late and to trigger unrecognized transmission chains [1],[3]. The sharp increase in immigration from high endemic countries is especially perceived as a cause of concern. Nevertheless, several studies conducted in some low-endemic European countries, including Italy, and a recent systematic review provide consistent evidence that TB in a foreign-born population does not significantly influence TB in the native population, although it contributes to increase the total number of cases of disease [2],[4]-[8].

In recent years immigration to Sicily, Italy, is increasing, often under the form of acute waves from the northern African shores. However, Sicily represents for most immigrants an intermediate step in their travel towards more economically appealing European countries. Consequently, the number of immigrants living in Sicily has raised from 24 900 in 1991 to about 140 000 in 2013, but their prevalence on the overall regional population is just 2.8% [9]. In 2013 Palermo and its province has registered about 29 000 resident immigrants, roughly doubling the proportion reported at the beginning of 2000s. Some demographic features are of special interest, such as a) the increase in the Romanian component, mainly following the accession to the EU since 2007, this community having become the main one in the island (about 20% of overall immigrants); b) the persistence of older established communities such as, for instance, Tunisians, Moroccans, Sri Lanka and Albanians; c) the increasing heterogeneity of the countries of origin, now accounting for more than 50, from the African continent to Latin America, from Asia to East Europe [10]. In the decade 1999-2008, according with the data provided by the Italian Health Ministry, the TB incidence in Sicily averaged 2.6 (0.9-3.5) per 100 000 population [11]. In 2008, in particular, the raw incidence of TB among foreign-born inhabitants was 11.2 per 100 000 compared to 0.7 per 100 000 in the autochthonous population. However, underreporting is very likely when considering that in the years 2003-2008 in Italy the incidence per 100 000 population declined from 5.1 to 3.8 in the Italian born inhabitants and ranged between 49.3 and 51.9 in the foreign born inhabitants [11].

Molecular epidemiology is increasingly used as a complementary tool to conventional epidemiology. Large “spoligotype” databases, describing the worldwide diversity of *Mycobacterium tuberculosis* complex (MTBC) clustered regularly interspaced short palindromic repeats (CRISPR) loci, have been released, allowing local epidemiological findings of MTBC to be studied in comparison with the global landscape [12]. Combining spoligotyping and 24 mycobacterial interspersed repetitive unit (MIRU)-variable-number tandem-repeat (VNTR) loci analysis has now replaced the traditional IS*6110*-restriction fragment length polymorphism technique in molecular epidemiology [13].

Only a limited number of data are available about the molecular epidemiological characteristics of MTBC in Italy [6],[7]. In the present study, we aimed to investigate the molecular epidemiological pattern of MTBC isolates in the province of Palermo, Sicily, by characterizing a set of isolates identified in the years 2004-2012 using spoligotyping and the 24-loci MIRU-VNTR method. A comparison with the findings obtained by restriction fragment length polymorphism (RFLP) typing of the IS*6110* insertion sequence and spoligotyping on a sample of 104 MTBC strains identified in the same geographic area in the years 1994-2000 [14],[15] is also presented.

## Methods

### MTBC isolates

**Figure 1:**
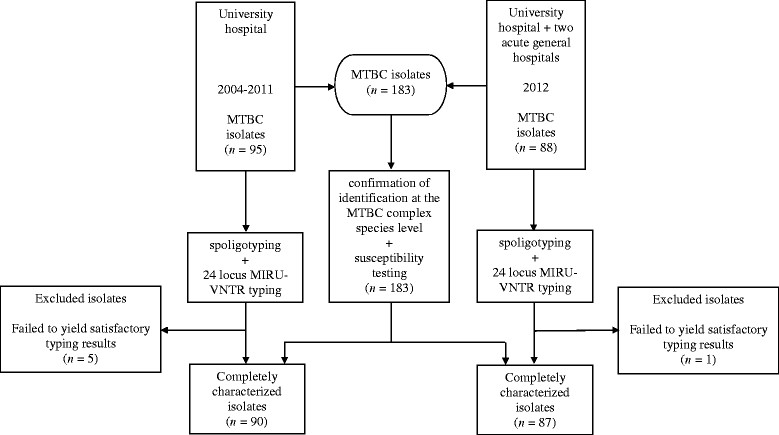
**Flowchart of the study describing source of MTBC isolates and typing methods.**

One hundred eighty-three MTBC isolates were analyzed (Figure 1). In particular, 95 isolates were prospectively collected during the year 2012 from patients admitted to the three largest acute general hospitals in Palermo, Italy. More specifically, the TB patients had been admitted to the Infectious Disease Unit of the University teaching hospital “Azienda Ospedaliero-Universitaria “Paolo Giaccone” and to the Infectious Disease and Respiratory Disease Units of the ARNAS Civico and Benfratelli hospital and Villa Sofia-V. Cervello hospital. The isolates accounted for about 90% of the isolates from laboratory-confirmed cases of TB identified in 2012 in the province of Palermo. Eighty-eight additional isolates were retrospectively recovered from a collection of MTBC isolates identified from patients admitted to Infectious Disease Unit of the University teaching hospital in the years 2004-2011. They accounted for approximately 13% of the isolates from laboratory-confirmed cases of TB in the province of Palermo in the same interval of time. MTBC isolates were obtained from both pulmonary (n = 168) and extra-pulmonary (n = 15) sites of infection.

Isolation had been carried out by routine methods on mycobacterial growth indicator tubes (Bactec MGIT 960; Becton Dickinson, Sparks, MD, US) and Lowenstein-Jensen slants. identification of MTBC isolates to the species level had been performed by a gene probe assay, according to the manufacturer's protocol (GenoType MTBC; Hain Lifescience GmbH, Nehren, Germany). Shortly after identification, the isolates had been stored at -70°C. For the study purposes, MTBC isolates were newly inoculated in MGIT and, subsequently, onto Lowenstein-Jensen slants and, after about 4-6 weeks of incubation at 35°C, when an abundant growth was apparent, all colonies were carefully suspended into 500 μl of Tris-EDTA buffer, pH 8.0, and heated for 20 min at 80°C. Mycobacterial genomic DNA was extracted by the QIAamp DNA Mini Kit (Qiagen, Germany). DNA was quantitated and checked for purity by NanoDrop® ND-1000 spectrophotometer (ThermoScientific, Wilmington, DE, US).

Susceptibility testing to streptomycin, isoniazid, rifampin and ethambutol was carried out at the Department of Sciences for Health Promotion and Mother-Child Care “G. D’Alessandro”, University of Palermo, Italy, using the mycobacterial growth indicator tube SIRE kit (Becton Dickinson, Sparks, MD, US).

Informed consent was not required to patients because the collection and typing of isolates followed their isolation and identification as a part of the usual patient care and no additional samples were necessary. All MTBC isolates were anonymized before their handling for the purposes of this study.

### Molecular typing

Spoligotyping assays were performed as described previously using a microbead-based DNA array method at the Institut de Génétique et Microbiologie UMR8621 CNRS-University Paris-Sud [16]. The 24-loci MIRU-VNTR method was performed using an electrophoretic gel based method in Palermo, Italy [17]. All results were transferred into Excel spreadsheet files and imported into the Bionumerics v. 6.4 software (Applied Maths, St Martins Latem, Belgium).

Genotypic lineage/sublineages were defined by entering the genotyping results into the latest publicly available SITVITWEB (http://www.pasteur-guadeloupe.fr:8081/SITVIT_ONLINE/). Clusters were defined as groups of patients infected with *M. tuberculosis* strains showing identical combined MIRU-VNTR and spoligotyping patterns.

Furthermore, the spoligotyping dataset obtained from the MTBC isolates identified in the years 1994-2000 [15] was reanalyzed in the light of the updated genetic diversity database, by merging it into the SITVITWEB database. The updated *tags* were used to newly label the previously characterized isolates. Minimum Spanning trees (MSTs) using spoligotyping data were computed by the Bionumerics software according with the manufacturer's instructions.

### Statistical analysis

The statistical analysis was carried out using the EpiInfo software (ver. 7.0.9.7), Centers for Disease Control and Prevention, Atlanta, GA, US. Means and frequencies were calculated and the significance of differences was assessed by one-way ANOVA test or Kruskall-Wallis, when appropriate, or by the chi-square test or the Fisher's exact test, respectively. The associations between the variables under examination were evaluated using contingency tables. All reported P values were two-sided and P < 0.05 was considered significant.

## Results

### TB patients - years 2004-2011 and 2012

Among the 95 TB cases identified in the period 2004-2011, 53 (55.8%) were Italian-born and 42 (44.2%) foreign-born individuals. The gender ratio was 2.06 (64 men, 31 women). The median age was 44.5 (interquartile range [IQR] 32.0-62.0) years for Italian-born patients and 34.0 (IQR 25.5-46.0) years for immigrant patients, P = 0.002.

The proportion of foreign-born patients among the 88 culture-proven TB cases identified in 2012 was 42.1% (37 foreign-born vs. 51 Italian-born patients). The gender ratio was 2.03 (59 men, 29 women). In this group of patients the median age was significantly higher in the Italian-born patients than in the foreign-born patients (Italian-born, median 57.5, IQR 40.0-71.0, years vs. foreign-born, median 34.5, IQR 29.5-46.5, years, P < 0.001).

There was not any statistically significant difference between the patients identified in 2004-2011 and 2012 with respect to their demographic characteristics, except for the significantly older median age of the Italian patients of the year 2012 compared to those of the period 2004-2011 (P = 0.04).

The country of birth was known for 32 out of 38 foreign-born TB cases bacteriologically-confirmed in 2012, but only in six out of 41 TB patients whose isolates had been collected in the years 2004-2011. Of the 38 foreign-born patients with known geographic origin, 17 were from Africa, 10 from Asia, nine from East Europe and two from Central America.

### Molecular typing of MTBC isolates

One hundred seventy-seven out the 183 isolates were completely characterized by spoligo- and 24-loci MIRU-VNTR typing (Figure 1). Because the two TB patients groups of 2004-2011 and 2012, respectively, had similar distribution by country of birth (Italian-born vs. foreign-born), the results of molecular typing of isolates were pooled and referred to the entire period 2004-2012.

**Figure 2:**
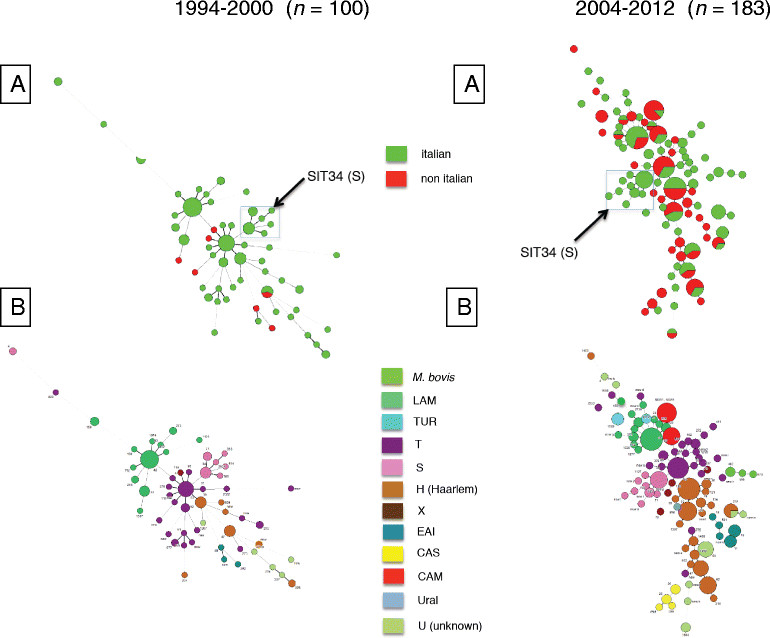
**Minimum Spanning Trees obtained from the spoligotyping dataset including 100 MTBC isolates identified in the period 1999-2004 and 183 MTBC isolates identified in the period 2004-2012. A**: by country of birth (Italian/non Italian). **B**: by main genotype families, Arrows indicate the “S” family.

Table 1 and Figure 2B summarize the distribution of MTBC isolates into spoligotyping defined genotypic lineages and families/subfamilies. Eleven different lineages were identified with the most represented being the Haarlem lineage with 47 (26.5%) isolates, the T super-family with 34 (19.2%) isolates, the Latin American-Mediterranean (LAM) lineage with 24 (13.6%) isolates and the S lineage with 15 (8.5%) isolates. The distribution of isolates of the T and LAM families roughly reflected the proportion of Italian-born and immigrant patients (Italian-born, T lineage 70.6% and LAM lineage 71.0%), whereas within the Haarlem lineage isolates from immigrants were disproportionately represented (55.3%). The S lineage isolates were exclusively detected in Italian patients (Table 1 and Figure 2B). They were more frequent in 2012 than in the period 2004-2011 (nine out of 87, 10.3% vs. six out of 90, 6.7%). The patients from whom the S isolates had been identified were older, but not significantly, than the Italian patients affected by other than S MTBC isolates (median age 71, IQR 42-75, years vs. 50.5, IQR 35-66, years, P = 0.25).

**Table 1:** **Distribution of the** ***Mycobacterium tuberculosis*** **complex isolates identified in Palermo, Italy, in the years 2004-2012 by lineage, family/sub-family and geographic source of patients**

			No. of isolates	
Lineage	nr. of isolates	Family/sub-family	Italian-born	Foreign-born
Haarlem	47	H3	15	13
		H1	5	6
		H	1	5
		H3 variant	-	2
T	34	T1	12	9
		T3	3	1
		T	5	-
		T2	2	-
		T2-T3	1	-
		T5	1	-
LAM	24	LAM 9	10	4
		LAM 5	2	1
		LAM 1	1	1
		LAM variant	1	1
		LAM 3	1	-
		LAM 4	1	-
		LAM	1	-
S	15	S	15	-
CAM	14	CAM	8	3
		CAM variant	-	3
U	13	U	10	3
EAI	10	EAI2 Manilla	3	-
		EAI3 India	1	2
		EAI6 BGD1	-	2
		EAI1 SOM	-	1
		EAI5	-	1
CAS	6	CAS 1Delhi	-	3
		CAS 1Delhi variant	1	2
Turkish	5	Turkish variant	-	3
		Turkish	-	2
X	4	X1	1	1
		X2	1	-
		X3	1	-
Bovis	4	Bovis BCG	2	-
		Bovis	1	-
		Bovis 1	1	-
Ural	1	Ural 1	1	-

Four isolates were classified as *M. bovis*, of which two as *M. bovis* BCG (bacillus Calmette-Guérin) sub-family.

A total of 27 (15.2%) strains were grouped into 10 clusters including two to six isolates. Two of them, including isolates belonging to the T and S lineages, grouped only Italian patients, whereas five clusters containing isolates of T, CAM or Turkish lineage included only non Italian patients. The remaining three clusters, including isolates of Haarlem and LAM lineages, contained each an equal proportion of Italian and non Italian patients. Two clusters, including CAM and Haarlem isolates, respectively, contained only isolates identified in 2012. The median age of clustered patients was lower compared to those unclustered, but not significantly (clustered vs. unclustered patients, median age 36.5, IQR 27.0-53.0, years vs. 40.5, IQR 31.0-55.0, years, P = 0.67).

### Comparison with the MTBC isolates 1994-2000

When spoligotyping data available from 100 out of 104 MTBC isolates identified in the years 1994-2000 were reanalyzed by merging them into the SITVITWEB database, they were attributed with seven previously described spoligotype lineages. Lineages T (30.8%), LAM (29.8%), Haarlem (16.3%) and S (13.5%) were predominant (Table 2). In addition, five strains could not be linked to a previously described lineage and were classified as “undefined”. No MTBC isolates belonging to CAM, U, CAS, Turkish and Ural lineages were identified. Of interest, only six isolates from foreign-born patients were present in the 1994-2000 dataset (Table 2).

**Table 2:** **Distribution by lineage and geographic source of patients of the two sets of** ***Mycobacterium tuberculosis*** **complex isolates under study**

	Years 1994 - 2000			Years 2004-2012		
		No. of isolates			No. of isolates	
Lineage	Total No. of isolates (%)	Italian-born	Foreign-born	Total No. of isolates (%)	Italian-born	Foreign-born
T	32 (30.8)	30	2	34 (19.2)	24	10
LAM	31 (29.8)	30	1	24 (13.6)	17	7
Haarlem	17 (16.3)	16	1	47 (26.5)	21	26
S	14 (13.5)	14	-	15 (8.5)	15	-
T4	1 (0.9)	-	-	-	-	-
EAI	3 (2.9)	1	2	10 (5.6)	4	6
X	1 (0.9)	1	-	4 (2.3)	3	1
CAM	-	-	-	14 (7.9)	8	6
U	-	-	-	13 (7.3)	10	3
CAS	-	-	-	6 (3.4)	1	5
Turkish	-	-	-	5 (2.8)	-	5
Bovis	-	-	-	4 (2.3)	4	-
Ural	-	-	-	1 (0.6)	1	-
Undefined	5	5	-	-	-	-

Figure 2 shows the population structure based upon spoligotyping data of the MTBC isolates from 1994-2000 (Figure 2A) and 2004-2012 (Figure 2B) and allows for a comparison of the respective distribution by main genotype families and by the country of birth of patients. The prevalence of the Euro-American phylogeographic lineage, including the spoligotype families T, Haarlem, LAM, S and X, was decreasing from 91.3% among the old isolates to 70.1% among the most recent ones (P < 0.001). Yet, the more recent set of isolates appeared to be characterized not only by a significant reduction of the proportion of strains belonging to the Euro-American lineage associated with the emergence of new spoligotype families, but also by a rearrangement of the spoligotype families into the same lineage, with the Haarlem family becoming more prevalent than T and LAM and the S family declining from 13.5% to 8.5% (Table 2). Of interest, among the 2004-2012 MTBC isolates, the previously undetected CAM, U, CAS, Turkish and Ural lineages accounted for 22.0% of isolates (Table 2).

### Drug resistance of MTBC isolates

Approximately 25% of the 183 MTBC isolates under study proved to be resistant to at least one antiTB drug with a higher prevalence within the isolates belonging to the U, S and T lineages (Table 3). The proportion of isolates resistant to one or more drugs did not significantly differ among foreign-born patients and Italian-born patients, respectively (21 out of 79 isolates, 26.6% vs. 24 out of 104 isolates, 23.1%, P = 0.58). The global prevalence of isoniazid resistance was 13.7%, with U and S isolates showing higher figures (Table 3). The proportion of isoniazid resistant isolates was significantly higher among the isolates from Italian-born patients, with a proportion more than twice as high as that from immigrant patients (19 out of 104 isolates, 18.3% vs. six out of 79 isolates, 7.6%, P = 0.04).

**Table 3:** **Antimicrobial drug resistance profiles of the** ***Mycobacterium tuberculosis*** **complex isolates by lineage, years 2004-2012, Palermo, Italy**

	Any drug resistance	Resistance to isoniazid	Multidrug resistance
Lineage	No. (%)*	No. (%)*	No.
U	5 (38.5)	4 (30.8)	2
S	5 (33.3)	3 (20.0)	-
T	8 (23.5)	4 (11.8)	-
Haarlem	9 (19.1)	3 (6.4)	-
LAM	2 (8.3)	2 (8.3)	1
CAM	7	3	-
Turkish	3	2	-
EAI	2	1	-
Bovis	2	2	-
CAS	1	1	-
ND	1	-	-
Total, No. 183	45 (24.6)	25 (13.7)	3 (1.6)

*percentage is showed only when the total number of isolates belonging to the lineage is >10.

Three MDR isolates were detected of which two belonging to the U lineage and one to the LAM9 sub-family. The first two isolates were from two elderly Italian patients, whilst the LAM9 isolate was from a 26-year old Eritrean patient.

## Discussion

The significant changes in TB epidemiology occurring in the last decades in low-incidence countries require to adopt fine-tuned investigation tools and to update strategies for TB control. In these settings, indeed, the analysis of transmission settings and dynamics is becoming increasingly complex due to ongoing striking socio-economic and demographic changes.

Sicily has a complex history of migration and cultural exchanges due to its strategic position at a crossroad of population movements involving Europe, North-Africa and the Eastern Mediterranean [18]. However, in the last two decades the migration events are dramatically changing both qualitatively and quantitatively, so putting together foreign-born long-term residents from traditional immigration countries with recently arrived migrants from an increasingly wide range of countries [10]. As a consequence, TB epidemiology is becoming a complex blend of reactivation in the older portion of the autochthonous people and in some groups of foreign-born individuals and recent transmission in the younger and more vulnerable population subgroups. Concentration of TB cases in hard-to-reach marginalized communities is a further issue of concern [1],[3],[8].

Our molecular epidemiology study highlights some interesting epidemiological features of TB in Palermo and its province. The first is the wide heterogeneity of the MTBC strains detected in the years 2004-2012 with the identification of 11 lineages and 35 families-subfamilies. For comparison, in another region of Italy, Tuscany, 10 lineages were identified among 1080 MTBC isolates identified in the years 2002-2005 [6]. This feature is even more evident when comparing the more recent distribution with that of the years 1994-2000, when seven lineages could be identified after reanalyzing the old spoligotyping dataset. Not only was the recent number of lineages higher, but the distribution of isolates was significantly different between the two consecutive periods mirroring the increasing contribution of isolates from foreign-born TB patients. Some lineages, consequently, such as CAM, CAS, Turkish and URAL were only detected in the second set of isolates. Moreover, the reciprocal trend of the declining T and LAM lineages vs. the increasing Haarlem lineages was likely attributable to the impact of isolates from immigrant patients. Interestingly, 10 isolates belonging to the EAI lineage were also identified and four of them were from Italian-born patients. The total prevalence of this lineage as well as its frequency among Italian patients were higher compared to previous reports from other Italian regions [6],[19]. The autochthonous S lineage was exclusively detected among Italian-born patients, according with previous reports [6],[7],[19].

It was an interesting finding the detection among the MTBC isolates under study of four *M. bovis* isolates, of which two *M. bovis* BCG, from Italian-born patients. Many southern Italy regions, including Sicily, are not still officially declared bovine tuberculosis free [19]. Consequently, possible acquisition of *M. bovis* through raw milk and raw milk products or through direct contact with infected animals cannot be ruled out. On the other hand, the presence of *M. bovis* BCG infections in Italians, which has been previously reported in other Italian regions, could be tentatively traced to the BCG treatment of bladder cancer patients [7]. However, no epidemiological data were available to test these hypotheses.

Our data also show some interesting epidemiological features of the clustered isolates. Four clusters including only non Italian TB patients were attributed with the newly detected lineages CAM and Turkish, whereas the more consolidated lineages Haarlem and LAM characterized the three clusters including isolates from both Italian and non Italian TB patients. The intrinsic limits of our isolate collection does not allow for drawing meaningful epidemiological inferences.

From the data in Table 2, it appears that 75.4% of the 183 MTBC isolates under study were susceptible to all anti-TB drugs tested, a lower proportion compared to previous Italian reports [6],[7]. The prevalence of isoniazid resistance and multidrug resistance is, on the contrary, similar to previously described figures in Italy and other low-incidence European countries [3],[6],[7]. The prevalence of resistance to at least one first line drug was similar among isolates from Italian-born and foreign-born patients. This finding, which is inconsistent compared with some previously reported observations about a higher prevalence of resistance among isolates from non-Italian patients in other Italian regions, could be related to the different patterns of countries of immigration and distribution of some MTBC lineages, such as the Beijing clade, which is both prevalent in some areas and frequently associated to antiTB drug resistance [6],[7].

A number of limitations should be considered when interpreting our results. The collection of MTBC isolates was likely not representative of the confirmed TB cases in the geographic area under investigation. In particular, the MTBC isolates identified in the years preceding 2012 were greatly underrepresented. The case mix of patients of the two periods, 2004-2011 and 2012, respectively, was likely different because of the different hospital specialty wards contributing to the collection of data and isolates. This probably skewed the age profile of the 2012 patients towards an older age group, so potentially influencing some features of the MTBC lineage distribution, such as the S lineage prevalence. Moreover, information about country of origin of the patients whom these isolates were recovered from was lacking, preventing us from associating more accurately molecular markers and geographic source. On the other hand, these limitations should have expectedly led to underestimate the genetic heterogeneity of the isolates under study which was conversely higher than that reported in more consistent MTBC collections of isolates [6],[7]. When comparing the lineage distribution between the two periods, 1994-2000 and 2004-2012, the results have to be interpreted cautiously because of the largely different composition of the two patients populations (6.0% vs. more than 40% MTBC isolates from foreign-born individuals). This ultimately skewed our comparison, though mirroring the impressive impact on TB epidemiology of the recent immigrant people influx in our geographic area. Clustering of isolates was also analyzed only for the purpose of comparing the two subsequent periods of study, but it could not provide information about possible recent transmission chains. Eventually, availability of conventional epidemiological data was limited.

## Conclusions

Molecular epidemiology of TB in Sicily is still embryonic. However, our data, though neither representative nor exhaustive, provide a picture of the molecular epidemiology of TB in Palermo, the largest province of Sicily, in the last years. The comparison between the present distribution of spoligotypes and that of about 10 years ago in the same geographic area suggests also how quickly and deeply the TB epidemiology has changed in a relatively short interval of time. This highlights the need for implementing an universal and continued laboratory-based surveillance of TB in Sicily with the aim to monitor indigenous transmission and import from high incidence countries and contribute to the assessment of prevention and control strategies.

## Authors’ original submitted files for images

Below are the links to the authors’ original submitted files for images. Authors’ original file for figure 1 Authors’ original file for figure 2

## Abbreviations

TB: Tuberculosis
ECDC: European Centre for Disease Prevention and Control
EU/EEA: European Union/European Economic Area
MTBC: *Mycobacterium tuberculosis* complex
CRISPR: Clustered regularly interspaced short palindromic repeats
MIRU: Mycobacterial interspersed repetitive unit
VNTR: Variable-number tandem-repeat
RFLP: Restriction fragment length polymorphism
MGIT: Mycobacterial growth indicator tube
IQR: Interquartile range
MDR: Multidrug resistant
