# Supplementary figures and images for: Molecular epidemiology of tuberculosis in Sicily, Italy: what has changed after a decade?
Source: BMC Infect Dis. 2014 Nov 19;14:602. doi: 10.1186/s12879-014-0602-4 (PMC4241219; doi:10.1186/s12879-014-0602-4)

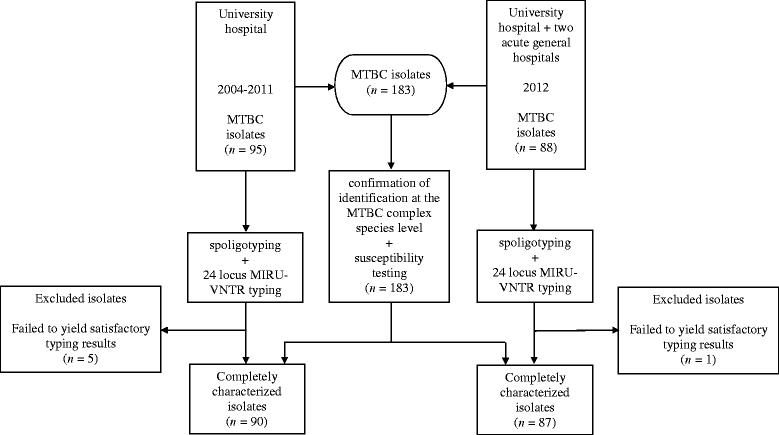

Supplement: Supplementary file 1 — Authors’ original file for figure 1 [file 12879_2014_602_MOESM1_ESM.gif]

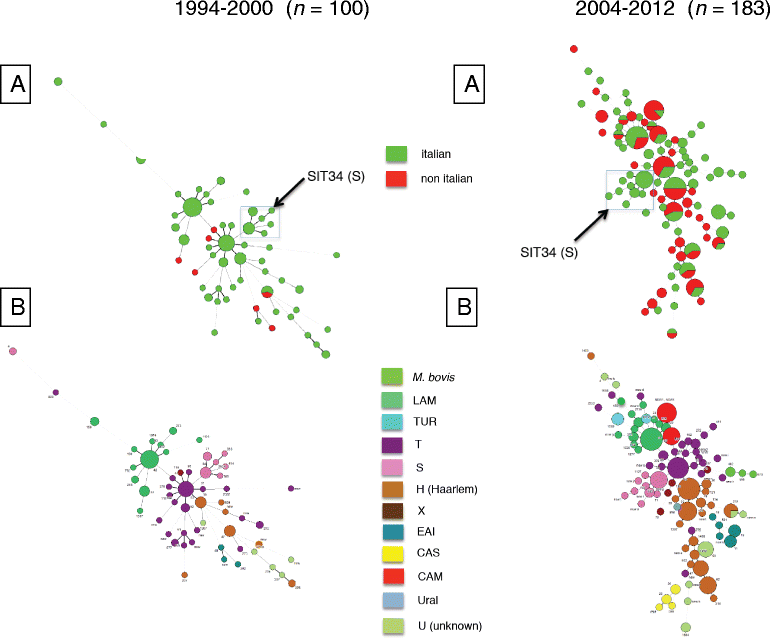

Supplement: Supplementary file 2 — Authors’ original file for figure 2 [file 12879_2014_602_MOESM2_ESM.gif]
